# Phosphonate inhibitors of West Nile virus NS2B/NS3 protease

**DOI:** 10.1080/14756366.2018.1506772

**Published:** 2018-10-26

**Authors:** Marcin Skoreński, Aleksandra Milewska, Krzysztof Pyrć, Marcin Sieńczyk, Józef Oleksyszyn

**Affiliations:** a Faculty of Chemistry, Division of Medicinal Chemistry and Microbiology, Wroclaw University of Science and Technology, Wroclaw, Poland;; b Faculty of Biochemistry, Biophysics and Biotechnology, Microbiology Department, Jagiellonian University, Krakow, Poland;; c Laboratory of Virology, Malopolska Centre of Biotechnology, Jagiellonian University, Krakow, Poland

**Keywords:** NS2B/NS3 protease, aminophosphonates, serine proteases, enzyme inhibitors, West Nile virus

## Abstract

West Nile virus (WNV) is a member of the flavivirus genus belonging to the *Flaviviridae* family. The viral serine protease NS2B/NS3 has been considered an attractive target for the development of anti-WNV agents. Although several NS2B/NS3 protease inhibitors have been described so far, most of them are reversible inhibitors. Herein, we present a series of α-aminoalkylphosphonate diphenyl esters and their peptidyl derivatives as potent inhibitors of the NS2B/NS3 protease. The most potent inhibitor identified was Cbz-Lys-Arg-(4-GuPhe)^P^(OPh)_2_ displaying *K_i_* and *k*_2_/*K_i_* values of 0.4 µM and 28 265 M^−1^s^−1^, respectively, with no significant inhibition of trypsin, cathepsin G, and HAT protease.

## Introduction

The West Nile virus (WNV) belongs to the *flavivirus* genus (*Flaviviridae* family) and is a mosquito-borne human pathogen of global occurrence. WNV was first isolated from humans in 1937 in the West Nile district of Uganda^1^. In 1953, it was identified in birds of the Nile delta region. Until 1997, WNV was not considered pathogenic to birds when a more virulent strain appeared in Israel and caused fatal disease with signs of encephalitis and paralysis in various bird species. In 1999, a pathogenic WNV strain was transferred to New York leading to its rapid spread throughout the USA, Canada and in the following years, the virus further spread, reaching northern countries of South America^2^. The virus also became a relevant human pathogen in Eurasia, causing large outbreaks in Greece, Israel, Romania, and Russia^3–6^. Although the lifecycle of WNV involves the transmission of viruses between birds and mosquitoes, various mammalian species, including humans, and horses, are susceptible to the virus. However, mammals are generally dead-end hosts, being infected through the bites of infected mosquitoes^7^. Although infections with WNV are mainly asymptomatic, one-fifth of the infected humans develops symptoms of the milder West Nile fever or more severe neuroinvasive diseases (meningitis and encephalitis). Unfortunately, no vaccine or effective antiviral therapy against WNV is available^8^.

The flaviviral genome is a positive-sense single strand RNA. The viral replication process occurs in the cytoplasm where the RNA serves as a template for production of a large polyprotein, which is further processed by host and viral proteases. This proteolytic maturation yields structural (C, prM, and E) and non-structural proteins (NS1, NS2A, NS2B, NS3, NS4A, NS4B, and NS5). NS3 plays a key role during the polyprotein processing. This protein is composed of an N-terminal protease domain (1–179 amino acids) and a C-terminal helicase domain (residues 180–618). It has been demonstrated that inactivation of NS2B/NS3 protease catalytic centre blocks viral replication^8^. To become fully functional, the NS3 segment requires a short co-factor, NS2B. The WNV protease contains the classical serine protease catalytic triad Asp-His-Ser. The protease binding site exists as a shallow groove composed of 7 subsites (S4-S3’, according to the Schechter and Berger nomenclature)^9^. An analysis of the substrate preference of WNV NS2B/NS3 protease revealed that the natural substrates contain a highly conserved arginine residue in the P_1_ position. Further studies showed that basic amino acids were also preferred in P_2_ as well as in the P_3_ positions^10^^,^^11^.

**Figure 1.**
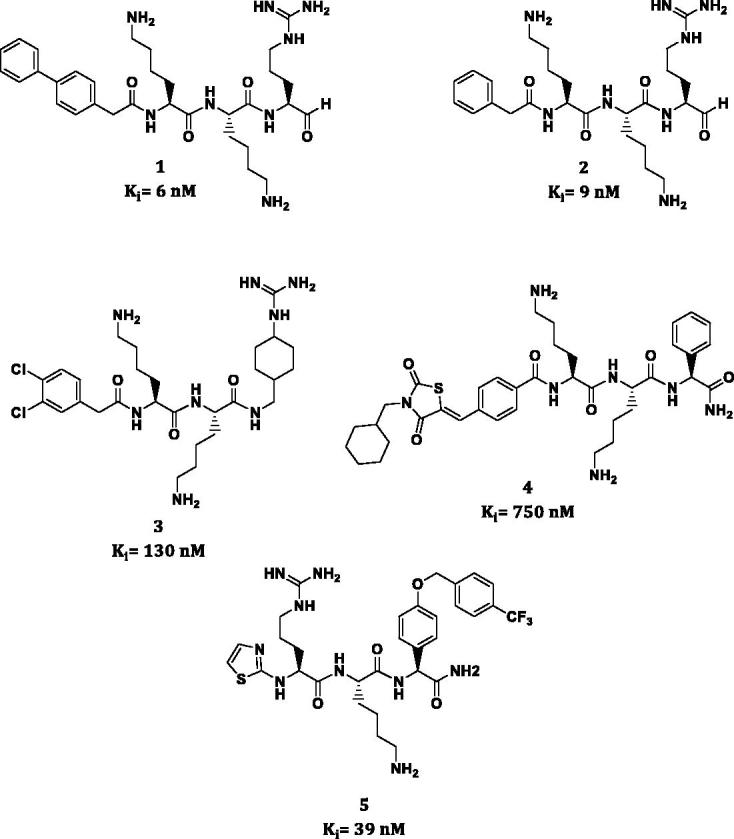
Inhibitors of the West Nile virus NS2B/NS3 protease.

Until now the most potent inhibitors of NS2B/NS3 protease have been reported by Stoermer et al.^11^. These compounds are tripeptide aldehydes (**1**,**2**) with a modified N-capping group (Figure 1). Although inhibitors **1** and **2** displayed low *K_i_* values of 6 and 9 nM, respectively, due to the high reactivity of an aldehyde group, low stability and tendency to form hemiaminals, their application as potential therapeutics is limited^12^. Hammamy et al. presented a series of decarboxylated substrate analogues containing chlorophenylacetyl (**3**) or phenylacetyl moiety as an N-capping group which are one of the most potent reversible NS2B/NS3 inhibitors reported thus far^13^. Recently, Bastos et al. presented an interesting group of novel peptide-hybrids reversible inhibitors based on 2,4-thiazolidinedione scaffold (**4**)^14^. An interesting reversible inhibitor of NS2B/NS3 was described by Behnam et al.^15^ compound **5** containing a benzyloxyphenylglycine residue at P1 position showed a significant reduction of Dengue and WNV titres in cell-based assays of virus replication (EC_50_ = 15.5 µM).

**Figure 2.**
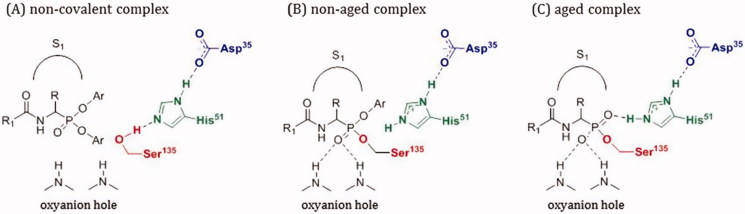
Mechanism of serine proteases inhibition by α-aminoalkylphosphonate diphenyl esters. Residue numbering according to the West Nile virus NS2B/NS3 protease.

Herein, we present the synthesis and application of α-aminoalkylphosphonates and their peptidyl derivatives as NS2B/NS3 WNV protease inhibitors. These compounds belong to a class of irreversible inhibitors that specifically and exclusively react with the active site serine residue leading to the formation of a slow hydrolysing protease-inhibitor complex (Figure 2)^16^. One of the major advantages of α-aminoalkylphosphonate diphenyl esters is their lack of reactivity with cysteine, aspartyl, and metalloproteases as well as good stability in buffer and human plasma^17^^,^^18^. In this work, we present a series of lysine, arginine, and peptidyl diphenylphosphonate derivatives which could be considered as starting templates for further structure optimisation studies as NS2B/NS3 protease inhibitors.

## Chemistry

### Chemical reagents

(Benzotriazol-1-yloxy)tripyrrolidinophosphonium hexafluorophosphate (PyBOP), CbzLys(Boc)-OH, Fmoc-Arg(Pbf)-OH, 2-chlorotrityl resin, trifluoroacetic acid, triisopropylsilane (TIPS), N,N-diisopropylethylamine (DIPEA), and di-*tert*-butyl dicarbonate (Boc_2_O) were purchased from IRIS Biotech (Marktredwitz, Germany). All other reagents, catalysts and solvents were purchased either from Sigma-Aldrich (Poznań, Poland), Merck (Warszawa, Poland), or Alfa Aesar (Karlsruhe, Germany).

### Inhibitors synthesis

The synthesis of simple Cbz-protected α-aminoalkylphosphonate diphenyl esters (**6–27)** was performed as previously reported^19,20^. For the overall synthetic strategy as well as the spectroscopic data of the obtained key intermediates and new products, see the Supplementary material associated with the manuscript. The spectroscopic data for already known compounds fully agreed with the literature data. Briefly, the synthesis of ornithine (**9**) and lysine (**6**) diphenylphosphonates started with the preparation of N-phthalimide-protected amino aldehydes, which were further used in the α-amidoalkylation reaction with benzyl carbamate and triphenyl phosphite (Scheme S1.A and S1.B)^20^. The phthalimide group of resulting orthogonally protected derivatives was removed with hydrazine hydrate, which led to the target compounds^21^. Their subsequent guanidinylation with *N*,*N^’^*-di-Boc-*S*-ethyl isothiourea in the presence of HgCl_2_ and triethylamine followed by Boc deprotection (50% TFA in DCM) resulted in diphenylphosphonate analogues of arginine (**7**) and homoarginine (**10**)^22^. In order to synthesise phosphonate analogue of thio-arginine (**8**), 4-chlorobutylaldehyde obtained under Swern conditions from 4-chlorobutane-1-ol was used in the amidoalkylation reaction with benzyl carbamate and triphenyl phosphite with Cu(CF_3_SO_3_)_2_ as a catalyst (Scheme S2)^23^. The obtained product was treated with thiourea in refluxing ethanol yielding target compound which crystallised as white solid from diethyl ether. The synthesis of diphenylphosphonate glutamine from 4-oxo-*N*-tritylbutanamide followed the procedure Ewa et al. (Scheme S3)^24^. The synthesis of 4-amino (**12**) and 4-guanidine (**13**) derivatives of diphenylphosphonate phenylalanine started from the esterification of 4-nitrophenylacetic acid with methanol followed by the reduction of nitro group (Scheme S4). After Boc protection of the amino group, the ester function was reduced to alcohol and then oxidised, by means of the Swern method. Subsequently, α-amidoalkylation with benzyl carbamate and triphenyl phosphite under mild conditions with Cu(CF_3_SO_3_)_2_ as catalyst produced Cbz-(4-*N*-Boc)Phe^P^(OPh)_2_. Boc deprotection with 50% TFA in DCM gave **12**, which was further transformed into **13** as described above (for derivatives **7** and **10**)^25^. A similar approach was applied for the synthesis of diphenylphosphonate phenylglycine derivatives (**14**–**19**) with the exception of nitrobenzaldehyde that was used for phosphonates synthesis, followed by nitro group reduction with SnCl_2_ prior to the guanylation step (Scheme S5)^26^. The synthesis of amidines **21** and **23** from the corresponding nitriles (**20** and **22**) followed the protocol developed by Oleksyszyn et al. (Scheme S6 and S7)^20^^,^^27^. Heterocyclic derivatives of diphenylphosphonate phenylglycine analogue (**24**–**27**) were obtained *via* the original α-amidoalkylation reaction procedure with substituted benzaldehydes obtained from 4-fluorobenzaldehyde and appropriate heterocyclic secondary amine (Scheme S8): pyrazole (**24**), benzimidazole (**25**), N-methylpiperazine (**26**), or morpholine (**27**)^20^^,^^28^.

**Figure 3.**
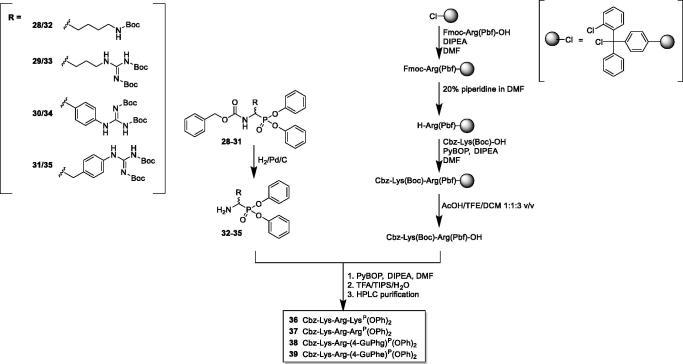
Synthesis of peptidyl α-aminoalkylphosphonate diphenyl esters (**35**–**38**) of general formula Cbz-Lys-Arg-Aaa^P^(OPh)_2_, where Aaa^P^ is a phosphonate ester analogue of arginine.

For the Cbz-protected derivatives that are most active against NS2B/NS3 protease, we extended their structure with a dipeptidyl Cbz-Lys-Arg fragment. The synthetic strategy, outlined in Figure 3, started with the removal of the Cbz protecting group in orthogonally protected phosphonates *via* hydrogenation over 10% Pd/C, yielding target derivatives (**32**–**35**) containing a free amino group. In parallel, the Cbz-Lys(Boc)-Arg(Pbf)-OH was synthesised using solid phase peptide synthesis approach on 2-chlorotrityl resin. Next, the obtained phosphonate analogues of arginine were coupled to Cbz-Lys(Boc)-Arg(Pbf)-OH using PyBOP as the coupling agent in the presence of DIPEA. The reaction was performed in DMF for 12 h. The reaction mixture was then diluted five times with ethyl acetate and washed with 5% citric acid, 5% NaHCO_3_, and brine. The organic phase was dried over anhydrous MgSO_4_, filtered and evaporated to dryness. The obtained crude product was treated with cleavage solution (95% TFA, 2.5% TIPS, 2.5% H2O; *v*/*v*/*v*) for 2 h at room temperature prior to the precipitation of deprotected phosphonate peptides with diethyl ether. The final compounds were purified on the HPLC (Varian ProStar 210 with a dual *λ* absorbance detector system equipped with the Discovery^®^ BIO Wide Pore C8 HPLC Column 250 mm × 212 mm, 10 µm) with a 15 ml/min flow rate using a gradient 5–95% (0.05% TFA/acetonitrile) in (0.05% TFA/H_2_O) over 15 min (Method A) or Discovery^®^ BIO Wide Pore C8 HPLC Column (250 mm × 46 mm, 10 µm) with a 0.9 ml/min flow rate using a gradient 0 − 100% (0.05% TFA/acetonitrile) in (0.05% TFA/H_2_O) over 15 min (Method B)). The nuclear magnetic resonance spectra (^1^H and ^31^P) were recorded on either a Bruker Avance DRX-300 (300.13 MHz for ^1^H NMR, 121.50 MHz for ^31^P NMR) or Bruker Avance 600 MHz (600.58 MHz for ^1^H NMR, 243.10 MHz for ^31^P NMR) spectrometer. Chemical shifts are reported in parts per million (ppm) relative to a tetramethylsilane internal standard. High-resolution mass spectrometry was acquired on Waters Acquity Ultra Performance LC, LCT Premier, XE.

### *In vitro* NS2B/NS3 protease inhibition

For the initial screening, Cbz-protected aminophosphonates were assayed in 96 well microplates (Nunc™ F96 MicroWell™ White Polystyrene Plate) in the following protease buffer: 50 mM Tris, 1 mM Chaps, 20% glycerol, pH 8.5. WNV NS2B/NS3 protease (AnaSpec, Liege, Belgium; 20 nM) was pre-incubated with tested inhibitor (100 µM) in the protease buffer at 37 **°**C for 10 min prior an addition of the fluorescent substrate Pyr-RTKR-AMC (AnaSpec, Liege, Belgium; 20 µM). The progress of the reaction was monitored continuously (*λ*_ex_ = 354 nm, *λ*_em_ = 442 nm) at 37 **°**C on a Spectra Max Gemini XPS spectrofluorometer (Molecular Devices, Sunnyvale, CA) for 30 min. For compounds which exhibited more than 50% of inhibition, the *K_i_* and *k*_2_/*K_i_* values were calculated. In 96 well microplates narrowed concentration of inhibitors and constant substrate concentration (Pyr-RTKR-AMC, C = 20 µM, K**_M_** = 59 µM) were prepared. The enzyme solution was added and enzymatic reaction was monitored (Figure S1). Using a model for irreversible inhibition, in which the first order inactivation rate constant k**_obs_** is hyperbolic dependent from the inhibitor concentration *K_i_* and *k*_2_/*K_i_* values were calculated (equations (1–4) in supplementary material)^29^. Control progress curves in the absence of inhibitor were linear. The standard deviation for the presented values was calculated using the mean of two independent experiments and did not exceed 10%.

### Control proteases inhibition assay

Bovine β-trypsin (AppliChem, Łódź, Poland), human cathepsin G (Biocentrum, Kraków, Poland) and Human Airway Trypsin-like Protease (HAT) (R&D Systems, Minneapolis, MN) were used as control proteases to screen the activity of NS2B/NS3 protease inhibitors (**36**–**39**) obtained in this study. Inhibitors were assayed in 96-well microplates in the 0.1 M HEPES, 0.5 M NaCl, 0.03%, pH 7.5 Triton X-100 (for bovine β-trypsin and cathepsin G). Bovine β-trypsin (15 nM), human cathepsin G (150 nM) or HAT protease (0.001 µg) was pre-incubated with tested inhibitor (25 µM) in the protease buffer at 37 °C for 10 min prior to the addition of the fluorescent substrate: Cbz-Arg-AFC (synthesised in-house according to the procedure described by Bissell^30^; 50 µM; *λ*_ex_ = 400 nm, *λ*_em_ = 505 nm, for bovine β-trypsin); MeO-Suc-Ala-Ala-Pro-Val-AMC (Bachem, Bubendorf, Switzerland; 40 µM, *λ*_ex_ = 340 nm, *λ*_em_ = 440 nm, for human cathepsin G) or Phe-Ser Arg-AMC (Bachem, Bubendorf, Switzerland; 40 µM, *λ*_ex_ = 340 nm, *λ*_em_ = 440 nm, for HAT protease). The progress of the reactions was monitored continuously at 37 °C on a Spectra Max Gemini XPS spectrofluorometer for 20 min. Control curves in the absence of inhibitor were linear. The rate of the tested protease inhibition was calculated from the linear range of the plot.

### Molecular docking

**Figure 4.**
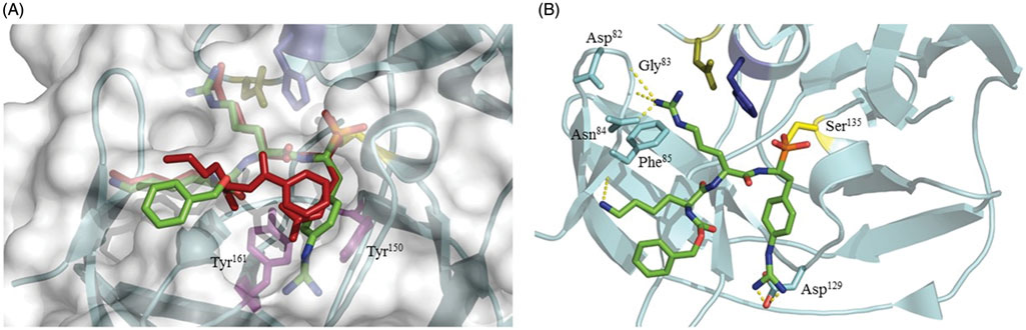
(A) Docking conformation of Cbz-Lys-Arg-(4-GuPhe)^P^(OH)_2_ (**38**, green) in the binding site of NS2B/NS3 protease. The peptidyl inhibitor is shown in red (Bz-Nle-Lys-Arg-Arg-H) is present in the original crystal structure of WNV protease (2*fp*7*.pdb*). (B) Interaction of Cbz-Lys-Arg-(4GuPhe)^P^(OH)_2_ (**38**, orange) with the NS2B/NS3 active site. The hydrogen bond network is indicated with yellow dashed lines.

In order to evaluate the binding mode of the obtained phosphonate inhibitors into the NS2B/NS3 active site, we have performed a molecular docking simulation (AutoDock Vina 1.1.2) using the WNV NS2B/NS3 protease (2fp7.pdb) as a receptor^31^. The coordinates of the Bz-Nle-Lys-Arg-Arg-H inhibitor molecule as well as water molecules were removed from the structure (Figure 4)^32^. Since numerous studies have shown that α-aminoalkylphosphonates inhibitors complex serine proteases as phosphonic acids, we docked energy minimised (MM2 force field) inhibitor **38** in the chemical form of (Cbz-Lys-Arg-(4-GuPhe)^P^(OH)_2_)^33^^,^^34^. The centre of the grid box was defined at the catalytic Ser hydroxyl oxygen with the grid box size 100 × 100 × 100 Å. Pictures were prepared in Pymol^35^.

## Results and discussion

### Inhibitor P*_1_* position screening

Compounds presented in this study are categorised into three groups: (I) compounds **6**–**11** are simple diphenyl phosphonate analogues of lysine, arginine, glutamine, ornithine, homoarginine, and thioarginine; (II) compounds **12**–**23** are aromatic analogues bearing a basic moiety, and (III) derivatives of diphenylphosphonatec phenylglycine (**24**–**27**) with different heterocyclic substituents. From all tested simple Cbz-N-capped derivatives, the highest potency of action toward NS2B/NS3 protease was observed for compounds **6**, **7**, **13**, and **16** (Table 1). The most potent compound was observed to be **13** with *k*_2_/*K_i_* value of 200 M^−1^s^−1^. Replacing the guanidine moiety in **13** with amino group (**12**) resulted in a dramatic drop in the inhibitory activity (11% of inhibition at 100 µM). In general, derivatives substituted at the *meta* position showed weaker inhibition levels as compared to their analogues substituted at *para* position of the phenyl ring (**14**, **15**, **16** vs. **17**, **18**, **19**). Compounds with heterocycles (**24**–**27**) showed very weak (1–5%) inhibitionagainst NS2B/NS3 protease when used at 100 µM concentration. This was probably because the heterocyclic group could not fit into the P1 binding pocket. The phenyl (**21**) and naphthyl (**23**) amidines were slightly more active against the tested protease. Nevertheless, among the tested series of α-aminoalkylphosphonate diphenyl esters we selected the most active compounds for further modifications. In summary, we identified lysine (**6**) and arginine (**7**) as the most favourable P_1_ residues, whereas for non-proteinogenic amino acid analogues we selected guanidine derivatives (**13**) and p (**16)**^23^.

**Table 1.** Activities of simple Cbz N-capped phosphonates against the NS2B/NS3 WNV protease.^a^

			

No	R	*K_i_* [μM]^b^	*k*_2_/*K_i_* [M^−1^s^−1^]
**6**		**22 ± 2 μM**	**80**
**7**		**13 ± 1 μM**	**154**
**8**		22%	
**9**		12%	
**10**		12%	
**11**		15%	
**12**		11%	
**13**		**4 ± 0.3 μM**	**200**
**14**		3%	
**15**		16%	
**16**		**10 ± 1 μM**	**87**
**17**		4%	
**18**		8%	
**19**		11%	
**20**		8%	
**21**		22%	
**22**		2%	
**23**		12%	
**24**		5%	
No	R	*K_i_* [μM]^b^	*k*_2_/*K_i_* [M^−1^s^−1^]
**25**		4%	
**26**		3%	
**27**		1%	

a Mean values ± standard deviation of two experiments conducted in duplicates.

b percent of inhibition was calculated for compounds which displayed low activity toward NS2B/NS3 protease after 30 min incubation at 37 °C; substrate used: Pyr-RTKR-AMC (C = 20 µM, K_M_ = 59 µM). Bold values indicate the most active compounds.

### Influence of peptide chain elongation

The structure of the most active inhibitors identified in the initial screening step was elongated with a P2 Arg and P3 Lys. The resultant compounds (**36**–**39**) showed significantly increased inhibitory potencies against NS2B/NS3 protease (Table 2). The introduction of the additional two residues into the structure resulted in a similar (69-fold) improvement in their inhibitory potency, leading to **36** (*k*_2_/*K_i_* = 5 520 M^−1^s^−1^) and **37** (*k*_2_/*K_i_* =  10 725 M^−1^s^−1^). The most potent NS2B/NS3 protease inhibitor identified in the presented studies was compound **38**, which displayed a *k*_2_/*K_i_* value of 28 265 M^−1^s^−1^. The highest (∼290-fold) increase of inhibitory potencies was observed for the peptidyl derivative of the phosphonate analogue of 4-guanidinephenylglycine (**39**) which showed a *k*_2_/*K*_i_ value of 24 890 M^−1^s. The inhibition data observed for **36**–**39** is in agreement with the reported X-ray structure of NS2B/NS3 protease, thus highlighting the significant role of P_2_ and P_3_ residues in binding molecules (substrates and inhibitors) to the enzyme^32^. As reference compound we synthesised peptide aldehyde inhibitor (**40**). This reversible inhibitor showed *K_i_* lower (∼4 times) than our most potent phosphonate inhibitor. However, it is difficult to compare the *K_i_* values between reversible and irreversible inhibitors. Noteworthy, all of the obtained compounds were tested as diastereoisomeric mixtures thus their separation into single isomers will lead to significantly more potent inhibitors as observed previously^36^. Further investigation into the design and synthesis of phosphonate inhibitors of NS2B/NS3 protease might lead to discovering more potent and selective inhibitors. Future work should involve structure-activity relationship studies of the P2 and P3 residues as well as ring substituents. The next challenge is a more comprehensive structure-activity relationship study aiming to optimise the peptidyl fragment of the inhibitor as well as the structure of the aromatic ring substituent prior to the *in vivo* studies of most potent derivatives.

**Table 2.** Activities of the peptide phosphonates against the NS2B/NS3 WNV protease^a^

No.	Compound	*K_i_* (μM)	*k*_2_/*K_i_* (M^−1^s^−1^)
**36**	Cbz-Lys-Arg-Lys^P^(OPh)_2_	8 ± 0.9	5 520
**37**	Cbz-Lys-Arg-Arg^P^(OPh)_2_	3 ± 0.3	10 725
**38**	Cbz-Lys-Arg-(4-GuPhe)^P^(OPh)_2_	**0.4 ± 0.03**	**28 265**
**39**	Cbz-Lys-Arg-(4-GuPhg)^P^(OPh)_2_	0.7 ± 0.2	24 890
**40**	Cbz-Lys-Arg-Arg-H	0.12 ± 0.02	Not determined

a Mean values þ standard deviation of two experiments conducted in duplicates. Bold value indicates the most active compound.

#### Protease selectivity assay

The selectivity of inhibitors **36**–**39** were determined by means of serine proteases of a similar substrate recognition pattern such as bovine β-trypsin, human cathepsin G, and HAT protease. The results clearly showed that the inhibition levels observed for all of the investigated compounds at a concentration of 25 µM did not exceed 10% after a 30 min incubation period (Table S2). These results indicate that the obtained peptidyl inhibitors are not significantly active against members of proteases with a trypsin-like activity. However, the selectivity toward other members of this family will be further examined.

### Molecular docking

The analysis of inhibitor **38** docked to the active site of NS2B/NS3 protease revealed docking conformation to be very similar to the one observed for Bz-Nle-Lys-Arg-Arg-H present in the crystal structure reported by Erbel et al. (2fp7.pdb)^32^. The reactive phosphonate warhead of the inhibitor is in close proximity (1.6 Å) to the catalytic serine residue allowing the formation of a covalent bond between the protease and inhibitor (Figure 4(A), Figure S3). The basic side chains of the amino acids in P_1_ and P_3_ positions are responsible for the formation of an extensive hydrogen bonding network with the protease (Figure 4(B)) including the P_1_ 4-guanidinephenylalanine electrostatic interaction with the side chain of Asp129; P_2_ arginine with the carbonyl oxygen of Gly83, and Asp82 and the side chain of Asn84; and lysine in P_3_ position with Phe85 carbonyl oxygen. Additionally, two tyrosine residues (Tyr150, Tyr161) could be responsible for interaction with aromatic ring of 4-guanidinephenylalanine inhibitor residue (Figure 4(A), Figure S4). This interaction provides explanation for the improved activity of inhibitors **38** and **39** over simple lysine and arginine analogues (**36,37**).

## Conclusions

In this work, we present a series of phosphonate diphenyl esters with low micromolar inhibitory activities against the WNV NS2B/NS3 protease. This class of inhibitors has never been reported to inhibit the NS2B/NS3 protease. The rigid 4-guanidinephenylalanine and 4-guanidinephenylglycine moieties at the P1 position were found to be more potent than a P1 arginine. Future work should involve more structure-activity relationship studies at the P2 and P3 residues and changing the phosphonate ester moieties.

## Supplementary Material

skorenski_supplementary.docx
