# Supplementary material for: Phosphonate inhibitors of West Nile virus NS2B/NS3 protease
Source: J Enzyme Inhib Med Chem. 2018 Oct 26;34(1):8–14. doi: 10.1080/14756366.2018.1506772 (PMC6211275; doi:10.1080/14756366.2018.1506772)
Supplement: skorenski_supplementary.docx [file IENZ_A_1506772_SM9333.docx]

**Supplementary materials**

**Phosphonate inhibitors of West Nile virus NS2B/NS3 protease**

Marcin Skoreński^a^, Aleksandra Milewska^b,c^, Krzysztof Pyrć^b,c^, Marcin Sieńczyk^a^, Józef Oleksyszyn^a^*

*^a^ Wroclaw University of Science and Technology, Faculty of Chemistry, Division of Medicinal Chemistry and Microbiology, Wybrzeze Wyspianskiego 27, 50-370 Wroclaw, Poland*

*^b^ Microbiology Department, Faculty of Biochemistry, Biophysics and Biotechnology, Jagiellonian University, Gronostajowa 7, 30-387 Krakow, Poland*

*^c^ Laboratory of Virology, Malopolska Centre of Biotechnology, Jagiellonian University, Gronostajowa 7, 30–387 Krakow, Poland*

*Corresponding author: jozef.oleksyszyn@pwr.edu.pl, phone 0048 71 320 40 27, fax 0048 71 320 24 27

**Scheme S1**. General strategy for the synthesis of Cbz-N-capped ornithine, lysine, arginine and homoarginine diphenylphosphonates.

**Scheme S2**. General strategy for the synthesis of Cbz-N-capped thioarginine diphenylphosphonate.

4-Chlorobutanal (**S2.1**). Oxalyl chloride (0.1 mol) was dissolved in freshly distilled dry dichloromethane (40 mL). The mixture was cooled to -60ºC and the solution of DMSO (0.27 mol) in dichloromethane (50 mL) was added dropwise. After 15 minutes 4-chlorobutan-1-ol (0.06 mol) dissolved in dichloromethane (50 mL) was added dropwise. The reaction was performed for 20 minutes and quenched by slow addition of trimethylamine (0.3 mol) followed by the addition of saturated ammonium chloride solution (100 mL). After the organic fraction was separated, aqueous phase was extracted with dichloromethane (3 × 50 mL). The combined organic fractions were washed with 5% citric acid, water and brine, dried over MgSO_4_, filtered and evaporated to dryness yielding final product (73%) as pale yellow oil. ^1^H NMRH NMR (300 MHz, CDCl_3_): *δ* 1.66-1.73 (m, 2H), 2.61-2.68 (m, 2H), 3.38-3.68 (m, 2H), 9.94 (s, 1H).

Benzyl (4-chloro-1-(diphenoxyphosphoryl)butyl)carbamate (**S2.2**). 4-chlorobutanal (0.037 mol), benzyl carbamate (0.037 mol) and triphenyl phosphite (0.037 mol) were dissolved in freshly distilled dry dichloromethane (20 mL) followed by an addition of copper triflate (0.004mol). The reaction was performed for 24 h at room temperature. Next, the volatile components of the reaction mixture were removed under reduced pressure. The resulting oil was dissolved in methanol and left for crystallization at -20 ºC yielding product (21%) as white solid. mp. 105-110ºC. ^31^P NMR (121 MHz, CDCl_3_): *δ* 18.68 (s). ^1^H NMRH NMR (300 MHz, CDCl_3_): *δ* 1.91-2.03 (m, 4H), 3.59 (t, *J* = 5.6 Hz, 2H), 4.44-4.57 (m, 1H), 5.10^*^ (d, *J* = 12.2 Hz, 1H), 5.16^*^ (d, *J* = 12.2 Hz, 1H), 5.28 (d, *J* = 10.1 Hz, 1H), 7.08-7.35 (m, 15H).

Benzyl (4-(carbamimidoylthio)-1-(diphenoxyphosphoryl)butyl)carbamate (**8**). Benzyl (4-chloro-1(diphenoxyphosphoryl)butyl)carbamate (0.001 mol) and thiourea (0.0012 mol) were dissolved in freshly distilled dry ethanol and the mixture was refluxed for 48 h. Next, the volatile components of the reaction mixture were removed under reduced pressure. The resulting oil was dissolved in minimal volume of methanol and diethyl ether was added. The solution was left for crystallization at 4 ºC yielding product (37%) as white solid. mp. 107 ºC. ^31^P NMR (121 MHz, DMSO-*d_6_*): *δ* 18.94 (s). ^1^H NMR (300 MHz, DMSO-*d_6_*): *δ* 1.74-1.98 (m, 4H), 3.58-3.60 (m, 2H), 4.19-4.26 (m, 1H), 4.97^*^ (d, *J* = 12.6 Hz, 1H), 5.03^*^ (d, *J* = 12.4 Hz, 1H), 6.89-7.33 (m, 19H), 8.02 (d, *J* = 9.6 Hz, 1H). ^13^C NMR (75 MHz, DMSO-*d_6_*): *δ* 26.53, 29.03 (d, *J* = 15.0 Hz), 45.09, 48.26 (d, *J* = 159.3 Hz), 66.40, 120.97 (dd, *J* = 3.7/16.1 Hz), 125.78 (d, *J* = 10.3 Hz), 128.30 (d, *J* = 13.2 Hz), 128.86, 130.37 (d, *J* = 5.4 Hz), 137.32, 150.29 (d, *J* = 9.3 Hz), 156.77, 184.36. MS (ESI):

514.1 (M+1).

**Scheme S3**. General strategy for the synthesis of Cbz-N-capped glutamine diphenylphosphonate.

**Scheme S4**. General strategy for the synthesis of Cbz-N-capped4-guanidinophenylalanine diphenylphosphonate derivatives.

**Scheme S5**. General strategy for the synthesis of Cbz-N-capped 4-guanidinophenylglycine diphenylphosphonate derivatives.

**Scheme S6**. General strategy for the synthesis of Cbz-N-capped 4-amidinophenylglycine diphenylphosphonate.

**Scheme S7**. General strategy for the synthesis of Cbz-N-capped4-amidinonaphthylglycine diphenylphosphonate.

6-(Methoxycarbonyl)-2-naphthoic acid (**S7.1**). Dimethyl naphthalene-2,6-dicarboxylate (0.02 mol) was heated to 80 ºC in dioxane (30 mL) until completely dissolved. Next, a solution of KOH (1.32 g) in methanol (10 mL) was added dropwise resulting in precipitation of white solid. The reaction as performed at 90 ºC for 2 h. White solid was filtered, washed with diethyl ether and dissolved in water (200 mL) followed by addition of 2M HCl until pH reached 3.0. Precipitated product was filtered, washed with water and dried on air yielding final product as white solid (83%). ^1^H NMR (300 MHz, DMSO-*d_6_*): *δ* 3.93 (s, 3H), 8.01-8.06 (m, 2H), 8.19-8.23 (m, 2H), 8.66 (s, 2H), 13.26 (s, 1H).

Methyl 6-carbamoyl-2-naphthoate (**S7.2**). Into the solution of 6-(methoxycarbonyl)-2-naphthoic acid (0.016 mol) in 1,2-dichloroethane (120 mL), thionyl chloride (60 mL) was added. The reaction mixture was refluxed for 3h until. Next, the volatile elements of the reaction mixture were removed under reduced pressure, redissolved in dry toluene (120 mL) and evaporated to dryness. The resulting oil was dissolved in dry dichloromethane (60 mL) and the solution of 7M ammonia in methanol (5 mL) was added. The reaction was performed at room temperature for 3h. The precipitated product was filtered yielding final product as white solid (93%). ^1^H NMR (300 MHz, DMSO-*d_6_*): *δ* 3.92 (s, 3H), 8.02 (d, *J* = 8.5 Hz, 2H), 8.11 (d, *J* = 8.6, 1H), 8.19 (d, *J* = 8.6 Hz, 2H), 8.55 (s, 1H), 8.66 (s, 1H).

Methyl 6-cyano-2-naphthoate (**S7.3**). Methyl 6-carbamoyl-2-naphthoate (0.01 mol) was suspended in dioxane (35 mL) followed by an addition of dry pyridine (2.4 mL). The reaction mixture was cooled in ice bath and trifluoroacetic anhydride (20 mL) was added dropwise. The reaction was let to equilibrate to room temperature. After 48h the reaction mixture was diluted with water (300 mL) and extracted with ethyl acetate (3×75 mL). The combined organic extracts were washed with water, dried over MgSO_4_, evaporated to dryness yielding product as white solid (44%). ^1^H NMR (300 MHz, DMSO-*d_6_*): *δ* 3.93 (s, 3H), 7.87 (dd, *J* = 1.6/8.5 Hz, 1H), 8.08-8.18 (m, 2H), 8.33 (d, *J* = 8.7 Hz, 1H), 8.65 (s, 1H), 8.72 (s, 1H). 6-(Hydroxymethyl)-2-naphthonitrile (**S7.4**). Methyl 6-cyano-2-naphthoate (0.008 mol) was dissolved in THF (100 mL) and LiBH_4_ (0.09 mol) was added. The resulting suspension was intensively stirred for 30 minutes followed by an addition of ethanol (100mL). The reaction was performed for at room temperature for 24h. Next, NaHCO_3_ (5% aq) was slowly added until the reaction mixture was transparent. The volatile components were removed under reduced pressure and the resulting aqueous fraction was extracted with CHCl_3_ (3×100 mL). Combined organic extracts were washed with water and brine, dried over MgSO_4_, filtered end evaporated to dryness yielding product as pale beige solid (96%). ^1^H NMR (300 MHz, CDCl_3_): *δ* 2.02 (s, 1H), 4.91 (s, 2H), 7.60 (dd, *J* = 1.3/8.5 Hz, 1H), 7.87-7.91 (m, 3H), 8.20 (s, 1H).

6-Formyl-2-naphthonitrile (**S7.5**) was synthesized using method described for S2.1 yielding target compound as pale yellow oil (95%), which was directly used in the next step.

Benzyl ((6-cyanonaphthalen-2-yl)(diphenoxyphosphoryl)methyl)carbamate (**22**). 6-Formyl-2-naphthonitrile (0.0055 mol), benzyl carbamate (0.0055 mol) and triphenyl phosphite (0.0055 mol) were dissolved in glacial acetic acid (6 mL). The reaction was performed at 80 ºC for 2h. The volatile elements were removed under reduced pressure and resulting oil was dissolved in methanol and left for crystallization at -20 ºC. The product was obtained as white solid (25%). ^31^P NMR (121 MHz, CDCl_3_): *δ* 14.53 (s). ^1^H NMR (300 MHz, CDCl_3_): *δ* 5.07^*^ (d, *J* = 12.1 Hz, 1H), 5.15^*^ (d, *J* = 12.3 Hz, 1H), 5.76 (dd, *J* = 9.1/22.8 Hz, 1H), 6.19 (q, *J* = 8.9 Hz, 1H), 6.87-7.37 (m, 15H), 7.59 (dd, *J* = 1.0/8.5 Hz, 1H), 7.72-7.88 (m, 3H), 7.99 (s, 1H), 8.19 (s, 1H,).

^13^C NMR (75 MHz, CDCl_3_): δ 53.02 (d, *J* = 157.4 Hz), 67.76, 110.08, 119.00, 120.31 (dd, *J* = 4.3/14.0 Hz), 125.61 (d, *J* = 6.6 Hz), 127.00, 127.42, 127.48, 127.57, 127.67, 128.27, 128.42, 128.60, 129.48 (d, *J* = 10.3 Hz), 129.82 (d, *J* = 8.1 Hz), 131.98, 133.83, 134.44, 135.39, 135.82, 149.91 (d, *J* = 9.6 Hz), 150.17 (d, *J* = 9.3 Hz), 155.68 (d, *J* = 11.4 Hz).

Benzyl ((6-carbamimidoylnaphthalen-2-yl)(diphenoxyphosphoryl)methyl)carbamate (**23**). Benzyl ((6cyanonaphthalen-2-yl)(diphenoxyphosphoryl)methyl)carbamate (0.9 mmol) was dissolved in the mixture of dry ethanol (1.7 mL) and dry chloroform (16 mL). The solution was saturated with gaseous HCl, sealed and kept at -20 ºC for 4 days. Next, diethyl ether (100 mL) was added and precipitated iminoether was filtered and dried in vacuum over P_2_O_5_. Next, collected iminoether was dissolved in methanol (50 mL) followed by an addition of 7M ammonia in methanol (0.15 mL). The reaction was performed at room temperature for 45 min prior evaporation of volatile components. Resulting oil was dissolved in methanol and refluxed for 8 h. The solvent was evaporated and the product was purified on Silica Gel (CHCl_3_:MeOH:AcOH/90:10:1; *v*/*v*/*v*) yielding final product as pale beige solid (29%). ^31^P NMR (121 MHz, DMSO-*d_6_*): *δ* 15.85 (s). ^1^H NMR (300 MHz, DMSO-*d_6_*): *δ* 5.06^*^ (d, *J* = 12.5 Hz, 1H), 5.16^*^ (d, *J* = 12.3 Hz, 1H), 5.83 (d, *J* = 22.8 Hz), 6.97-7.35 (m, 15H), 7.89 (dd, *J* = 8.4/22.1 Hz, 2H), 8.08 (t, *J* = 7.8 Hz, 2H), 8.27 (s, 1H), 8.44 (s, 1H), 10.37 (br s, 5H). ^13^C NMR (75 MHz, DMSO-*d_6_*): *δ* 50.89 (d, *J* = 155.2 Hz), 66.79, 120.73 (d, *J* = 3.8 Hz), 124.75, 125.85 (d, *J* = 6.3 Hz), 127.94, 128.44, 128.86, 129.04, 129.71, 130.35 (d, *J* = 3.8 Hz), 131.85, 134.79, 135.27, 137.09, 166.31, 177.20. MS (ESI): *m/z* = 566.8 (M^+^)


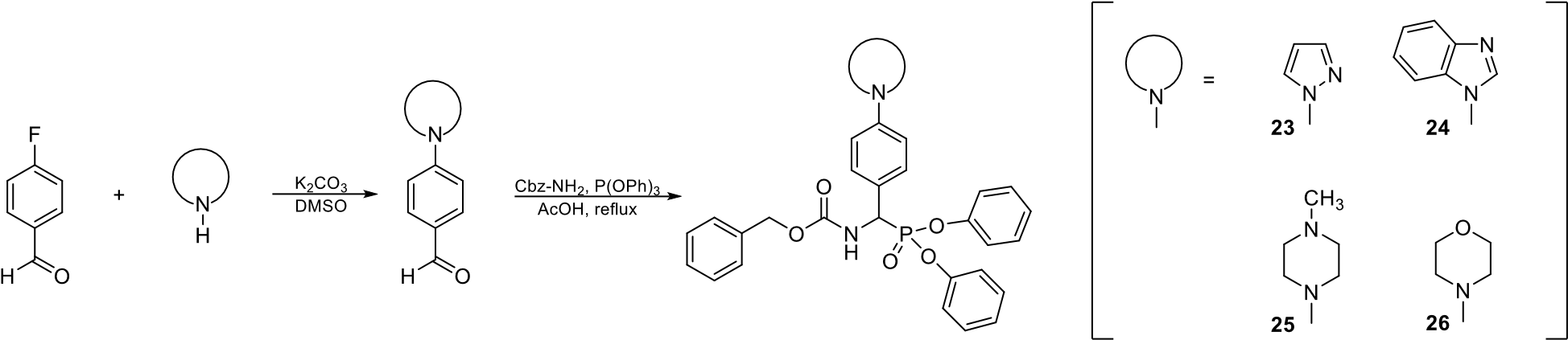


**27**

**26**

**25**

**24**

**Scheme S8**. General strategy for the synthesis of Cbz-N-capped 4-guanidinophenylglycine diphenylphosphonate derivatives.

Benzyl ((4-(1H-pyrazol-1-yl)phenyl)(diphenoxyphosphoryl)methyl)carbamate (**24**). Yield: 53%. ^31^P NMR

(121 MHz, CDCl_3_): *δ* 15.06 (s). ^1^H NMR (300 MHz, CDCl_3_): *δ* 5.05^*^ (d, *J* = 12.2 Hz, 1H), 5.13^*^ (d, *J* =

12.3 Hz, 1H), 5.61 (dd, *J* = 9.4/22.2 Hz, 1H), 6.05 (dd, *J* = 3.6/9.6 Hz, 1H), 6.47 (t, *J* = 1.8 Hz, 1H), 6.916.94 (m, 2H), 7.10-7.32 (m, 15H), 7.52-7.60 (m, 2H), 7.71-7.73 (m, 3H), 7.91 (d, *J* = 2.5 Hz, 1H). ^13^C NMR (75 MHz, CDCl_3_): *δ* 53.39 (d, *J* = 157.7 Hz), 67.66, 107.92, 119.37, 120.40 (dd, *J* = 4.1/8.9 Hz), 125.50 (d, *J* = 3.0 Hz), 126.75, 128.30 (d, *J* = 10.9 Hz), 128.60, 129.35 (d, *J* = 6.3 Hz), 132.37, 135.90, 140.35, 141.38, 150.00 (d, *J* = 10.0 Hz), 155.45 (d, *J* = 10.5 Hz).

Benzyl ((4-(1H-benzo[d]imidazol-1-yl)phenyl)(diphenoxyphosphoryl)methyl)carbamate (**25**). Yield: 18%. ^31^P NMR (121 MHz, CDCl_3_): *δ* 14.69 (s). ^1^H NMR (300 MHz, CDCl_3_): *δ* 5.16-5.25 (m, 2H), 5.74 (dd, *J* = 10.2/25.8 Hz, 1H), 6.30 (d, *J* = 7.2 Hz, 1H), 7.12-8.04 (m, 24H).

Benzyl ((diphenoxyphosphoryl)(4-(4-methylpiperazin-1-yl)phenyl)methyl)carbamate (**26**). Yield: 31%. ^31^P NMR (121 MHz, CDCl_3_): *δ* 16.02 (s). ^1^H NMR (300 MHz, CDCl_3_): *δ* 2.35 (s, 3H), 2.57 (t, *J* = 4.8 Hz, 4H), 3.21 (t, *J* = 4.7 Hz, 4H), 5.04^*^ (d, *J* = 12.1 Hz, 1H), 5.14^*^ (d, *J* = 12.1 Hz, 1H), 5.49 (dd, *J* = 10.1/21.7 Hz, 1H), 5.86 (d, *J* = 7.4 Hz, 1H), 6.83-7.38 (m, 19H). ^13^C NMR (75 MHz, CDCl_3_): *δ* 46.10, 48.68, 52.34 (d, *J* = 163.5 Hz), 54.98, 67.48, 116.02, 120.50 (t, *J* = 4.4 Hz), 124.54, 125.28 (d, *J* = 5.1 Hz), 128.27, 128.56, 129.13 (d, *J* = 6.7 Hz), 129.66 (d, *J* = 8.5 Hz), 151.45.

Benzyl ((diphenoxyphosphoryl)(4-morpholinophenyl)methyl)carbamate (**27**). Yield: 48%. ^31^P NMR (121 MHz, CDCl_3_): *δ* 15.88 (s). ^1^H NMR (300 MHz, CDCl_3_): *δ* 3.15 (t, *J* = 4.9, 4H), 3.85 (t, *J* = 4.7 Hz, 4H), 5.04^*^ (d, *J* = 12.2 Hz, 1H), 5.13^*^ (d, *J* = 12.2 Hz, 1H), 5.50 (dd, *J* = 10.1/21.6 Hz, 1H),5.89 (d, *J* = 8.4 Hz, 1H), 6.85-7.38 (m, 19H). ^13^C NMR (75 MHz, CDCl_3_): *δ* 49.01, 52.33 (d, *J* = 159.7 Hz), 66.82, 67.49, 117.71, 120.49 (t, *J* = 4.6 Hz), 125.01, 125.29 (d, *J* = 5.1 Hz), 128.24 (d, *J* = 6.3 Hz), 128.56, 129.18 (d, *J* = 6.6 Hz), 129.67 (d, *J* = 8.0 Hz), 150.20 (d, *J* = 9.6 Hz), 151.48, 155.40.

Benzyl ((2R)-6-amino-1-(((2R)-1-((5-amino-1-(diphenoxyphosphoryl)pentyl)amino)-5-guanidino-1oxopentan-2-yl)amino)-1-oxohexan-2-yl)carbamate (**36**). ^1^H NMR (400 MHz, DMSO-*d_6_*): *δ* 8.31 (d, *J* = 7.7 Hz, 1H), 7.78 – 7.62 (m, 4H), 7.69 – 7.58 (m, 1H), 7.55 – 7.33 (m, 4H), 7.30 – 7.24 (m, 4H), 7.18 – 7.07 (m, 6H), 6.77 – 6.71 (m, 6H), 5.12 – 4.95 (m, 2H), 4.41 – 4.30 (m, 1H), 4.23 – 4.11 (m, 1H), 4.07 – 3.88 (m, 1H), 3.21 – 2.97 (m, 4H), 2.74 – 2.63 (m, 2H), 1.82 – 1.75 (m, 2H), 1.72 – 1.62 (m, 2H), 1.65 – 1.36 (m, 11H), 1.30 – 1.09 (m, 4H).

. ^31^P NMR (243 MHz, DMSO-*d_6_*): *δ* 18.21 (s, 52%), 18.78 (s,48%). HRMS: calcd for (C_37_H_53_N_8_O_7_P)Na^+^, 775.3673; found, 775.3675. HPLC: t_R_ = 13.85 min.

Benzyl ((2R)-6-amino-1-(((2R)-1-((1-(diphenoxyphosphoryl)-4-guanidinobutyl)amino)-5-guanidino-1oxopentan-2-yl)amino)-1-oxohexan-2-yl)carbamate **(37)** ^1^H NMR (400 MHz, DMSO-*d_6_*) *δ* 9.33 (s, 1H), 8.20 (d, *J* = 7.5 Hz, 1H), 7.95 (d, *J* = 7.9 Hz, 1H), 7.74 – 7.65 (m, 4H), 7.64 – 7.58 (m, 1H), 7.42 – 7.37 (m, 1H), 7.36 – 7.24 (m, 8H), 7.14 – 7.08 (m, 5H), 6.74 – 6.68 (m, 7H), 5.06 – 4.89 (m, 2H), 4.30 – 4.23 (m, 1H), 4.17 – 4.08 (m, 1H), 4.00 – 3.91 (m, 1H), 3.16 – 2.98 (m, 4H), 2.77 – 2.61 (m, 2H), 1.76 – 1.64 (m, 2H), 1.62 – 1.52 (m, 2H), 1.52 – 1.42 (m, 9H), 1.34 – 1.17 (m, 3H).. ^31^P NMR (162 MHz, DMSO-*d_6_*): *δ* 18.15 (s, 54%), 18.72 (s, 46%). HRMS: calcd for (C_37_H_53_N_10_O_7_P)Na^+^, 803.3734; found, 803.3737. HPLC: t_R_ = 13.14 min.

Benzyl ((2R)-6-amino-1-(((2R)-1-((1-(diphenoxyphosphoryl)-2-(4-guanidinophenyl)ethyl)amino)-5guanidino-1-oxopentan-2-yl)amino)-1-oxohexan-2-yl)carbamate (**38**). ^1^H NMR (400 MHz, DMSO-*d_6_*): δ 10.18 (s, 1H), 8.85 (s, 1H), 8.14 (d, *J* = 7.8 Hz, 1H), 7.73 (s, 3H), 7.57 (s, 3H), 7.46 (s, 1H), 7.44 (s, 1H), 7.41 – 7.24 (m, 10H), 7.22 (d, *J* = 1.2 Hz, 1H), 7.21 – 7.15 (m, 4H), 7.12 – 7.04 (m, 5H), 5.01 – 4.93 (m, 2H), 4.54 – 4.38 (m, 1H), 4.19 – 4.12 (m, 1H), 4.04 – 3.88 (m, 1H), 3.41 – 3.30 (m, 2H), 3.29 – 3.15 (m, 2H), 3.11 – 2.98 (m, 2H), 2.78 – 2.65 (m, 2H), 1.78 – 1.66 (m, 1H), 1.63 – 1.53 (m, 2H), 1.52 – 1.42 (m, 6H), 1.39 – 1.25 (m, 3H). ^31^P NMR (162 MHz, DMSO-*d_6_*): *δ* 18.12 (s, 54%), 18.50 (s, 46%). HRMS: calcd for (C_41_H_53_N_10_O_7_P)Na^+^, 851.3734; found, 851.3740. HPLC: t_R_ = 14.91 min.

Benzyl ((R)-6-amino-1-(((R)-1-(((4-carbamimidoylphenyl)(diphenoxyphosphoryl)methyl)amino)-5guanidino-1-oxopentan-2-yl)amino)-1-oxohexan-2-yl)carbamate (**39**). ^1^H NMR (400 MHz, DMSO-*d_6_*): 10.08 (s, 1H), 8.71 (s, 1H), 8.11 – 8.07 (m, 1H), 7.81 – 7.72 (m, 7H), 7.45 – 7.21 (m, 11H), 7.15 (d, *J* = 1.5 Hz, 1H), 7.11 – 7.03 (m, 4H), 7.00 – 6.92 (m, 5H), 5.05 – 4.90 (m, 2H), 4.49 – 4.36 (m, 1H), 4.21 – 4.18 (m, 1H), 4.07 – 3.85 (m, 1H), 3.47– 3.41 (m, 2H), 3.31 – 3.22 (m, 2H), 2.80 – 2.71 (m, 2H), 1.82 – 1.72 (m, 1H), 1.65 – 1.51 (m, 2H), 1.48 – 1.40 (m, 6H), 1.34 – 1.21 (m, 3H).^31^P NMR (162 MHz, DMSO-*d_6_*) *δ* 18.18(s, 51%), 18.47 (s, 49%). HRMS: calcd for (C_40_H_50_N_9_O_7_P)Na^+^, 822.3469; found, 822.3477. t_R_ = 14.71 min.

**Scheme S9**. Synthesis of reference peptide aldehyde inhibitor (**40**)


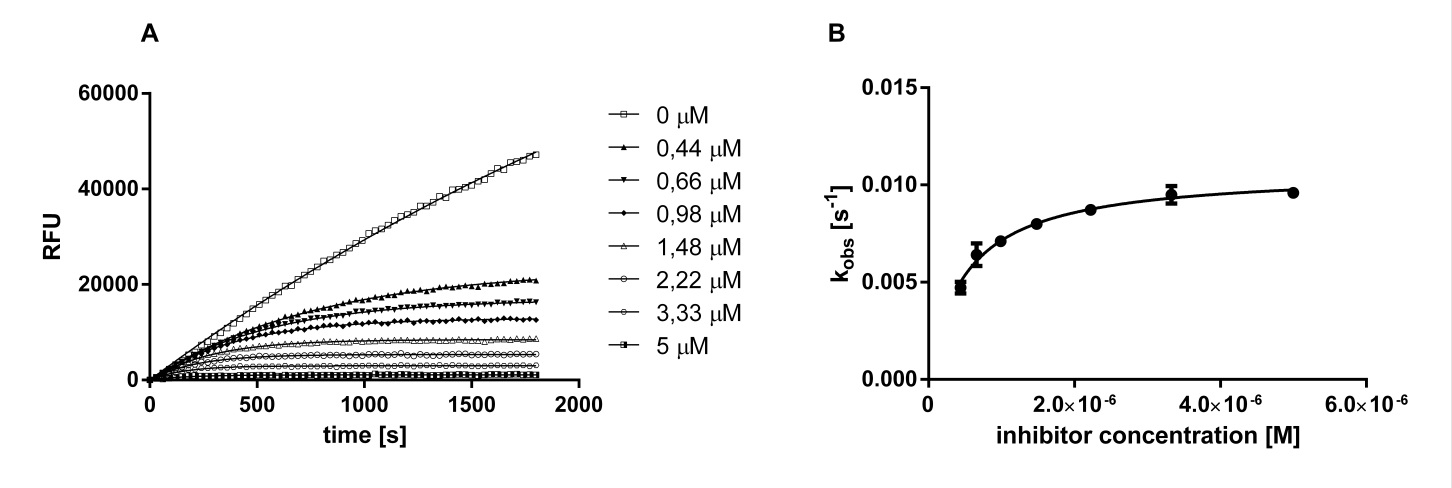


Figure S1. Enzyme (WNV NS2B/NS3) reaction progress curves with in the presence of inhibitor 38 in different concentrations (A). Plot of k_obs_ as function of inhibitor (38) concentration (B). Substrate used: Pyr-RTKR-AMC (C=20 µM, K_M_= 59 µM).

*Mathematical Inhibition Model.*

Mechanism of inhibitory action can be expressed as this simplified equation:

$E+I \begin{matrix} \underset{\to}{k_{1}} \\ \overset{\leftarrow}{k_{-1}} \end{matrix} E\cdot I \underset{\to}{k_{2}} E-I$

where K_i_ is the reversible enzyme–inhibitor complex dissociation constant, and k_2_ is the inactivation rate constant. Thus the k_2_/K_i_ value for irreversible phosphonic-type inhibitors fully reflects their potency of action towards the target serine protease.

The inhibitory activity of the compounds was determined by the progress curve method. First the data from nonlinear progress curves with time dependent inhibitors were fit to eq.1 to obtain the first-order rate constant (k_obs_):

$\left[ P \right]=v_{s}t+ \frac{v_{i}-v_{s}}{k_{obs}} [1-e^{\left( -k_{obs}t \right)}]$

(1)

[P] is the product concentration at time *t*, *v_i_* is the initial velocity, and *v_s_* is the steady state velocity

In case of irreversible inhibitors *v_s_* is zero thus equation (1) could be simplify to equation (2):

$\left[ P \right]=\frac{v_{i}}{k_{obs}} [1-e^{\left( -k_{obs}t \right)}]$

(2)

The second-order rate constants (k_2_/K_I_) and (K_I_)*_app_* values were calculated using hyperbolic regression according to equation 3:

$$k_{obs}=\frac{k_{2}[I]}{K_{I}{{(K}_{I})}_{app}+[I]}$$

(3)

k_obs_ is the first-order rate constant at known inhibitor concentration [I]

K_I_  values were calculated according to equation 3:

$$K_{I}=\frac{{{(K}_{I})}_{app}}{1+\frac{[S]}{K_{M}}}$$

*Stability in assay buffer.* A stock solution of compound (**38**) (10 mM in acetonitrile) was added to the assay buffer (50 mM Tris, 1 mM Chaps, 20% glycerol, pH 8.5) to make a final concentration of 2 mg/mL and incubated at 37°C. The resulting mixture was subjected to HPLC analysis to determine compound stability using a Discovery® BIO Wide Pore C8 HPLC Column (250 mm × 4.6 mm, 10 μm) with a 1 mL/min flow rate, gradient 0−100% [0.05% TFA in acetonitrile] in [0.05% TFA in water] over a period of 15 min.

Obtained results are presented in Table S1 and Figure S2. Over 6 hours we observed degradation of ~13% of compound **38.** However during enzyme inhibition assay time inhibitor degradation is almost unnoticeable (~1%). As we expect degradation product is corresponding monoester of compound **38**. This degradation process is probably caused by basic character of used buffer (pH 8.5).

Table **S1.** Stability of **38** in assay buffer (50 mM Tris, 1 mM Chaps, 20% glycerol, pH 8.5)

| Time[h] | % of initial inhibitor concentration |
| --- | --- |
| 0.0 | 100 |
| 0.5 | 98,93 |
| 1.0 | 98,12 |
| 1.5 | 96,27 |
| 2.0 | 95,19 |
| 2.5 | 95,04 |
| 3.0 | 93,35 |
| 3.5 | 91,90 |
| 4.0 | 91,11 |
| 4.5 | 88,43 |
| 5.0 | 86,99 |
| 5.5 | 86,84 |
| 6.0 | 86,73 |


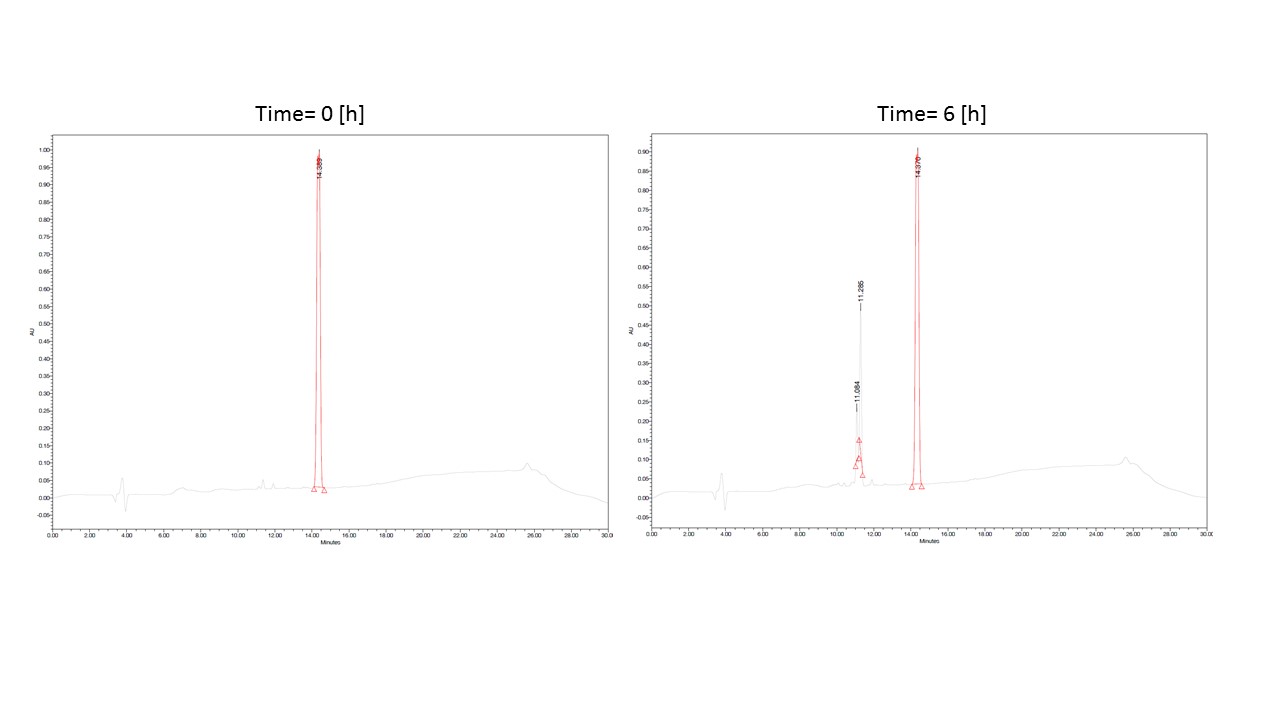


Figure S2. Chromatograms from stability of inhibitor 38 in assay buffer

Table **S2.** Activity of tested compounds against trypsin, cathepsin G and HAT protease.

| **No.** | **Compound** | **Trypsin** | **CatG** | **HAT** |
| --- | --- | --- | --- | --- |
| **36** | Cbz-Lys-Arg-Lys^P^(OPh)_2_ | 8%^a^ | 2% | 2% |
| **37** | Cbz-Lys-Arg-Arg^P^(OPh)_2_ | 7% | 3% | 2% |
| **38** | Cbz-Lys-Arg-(4-GuPhe)^P^(OPh)_2_ | 4% | 7% | 5% |
| **39** | Cbz-Lys-Arg-(4-GuPhg)^P^(OPh)_2_ | 5% | 9% | 6% |

^a^ percent of inhibition was calculated for compounds which displayed low activity toward protease after 30 min incubation at 37º C at inhibitor concentration 25μM


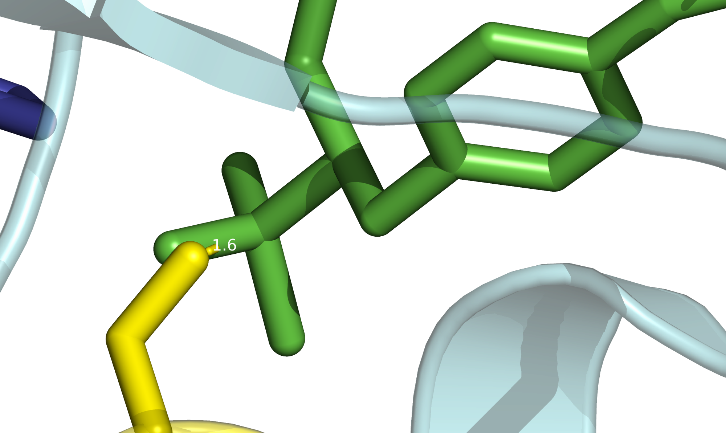


**Figure S3.** Localization of phosphonate (green) moiety near catalytic serine residue (yellow)


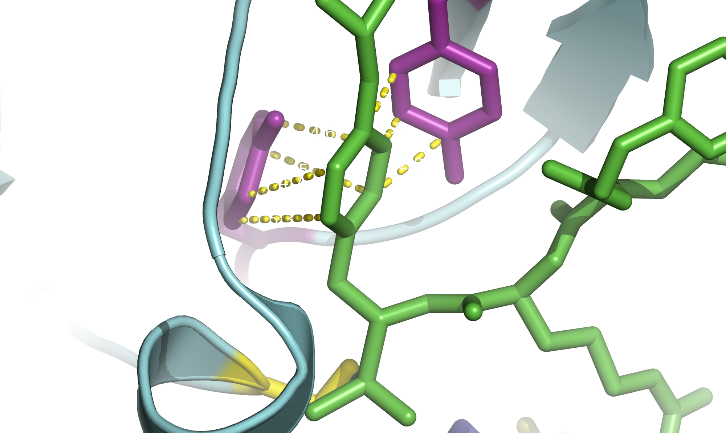


**Figure S.** Interactions of 4-guanidinephenylalanine moiety with enzyme tyrosine residues

**Figure S5.**^1^H NMR and ^31^P NMR of inhibitor **37**

**Figure S6.**^1^H NMR and ^31^P NMR of inhibitor **38**


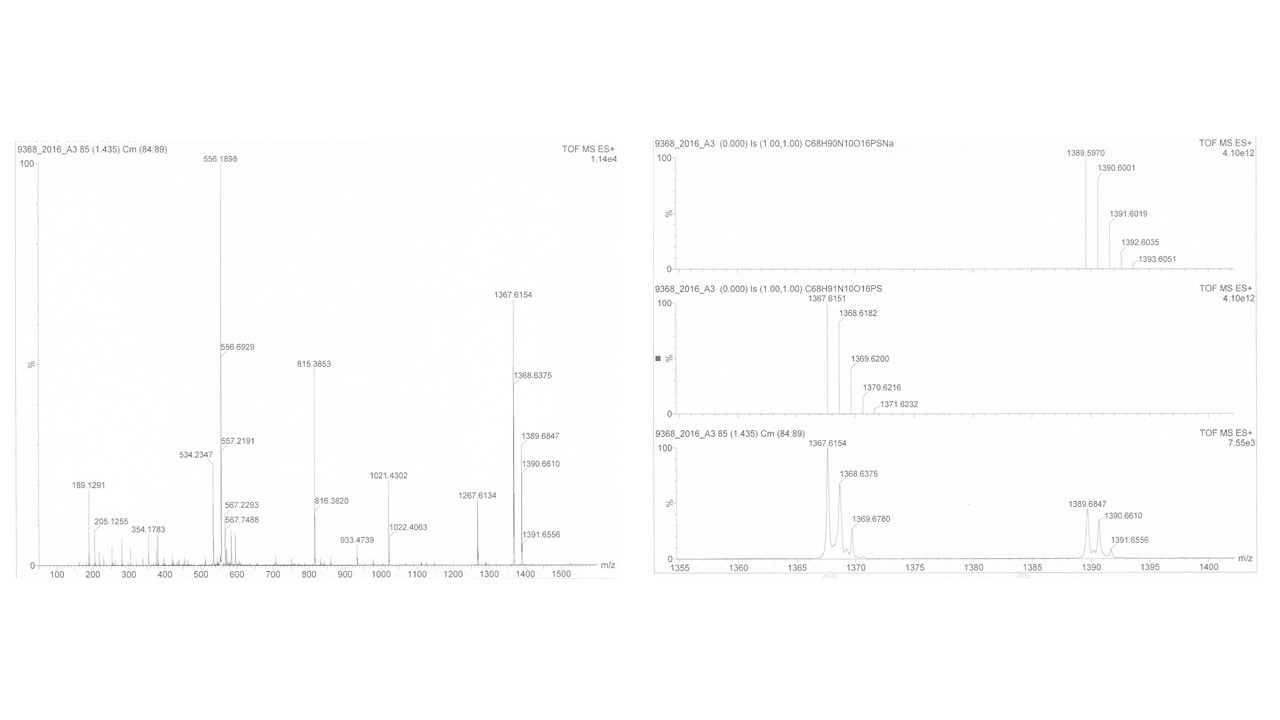


**Figure S7.** HRMS spectra from coupling reaction of **34** with dipeptide Cbz-Lys(Boc)-Arg(Pbf)-OH


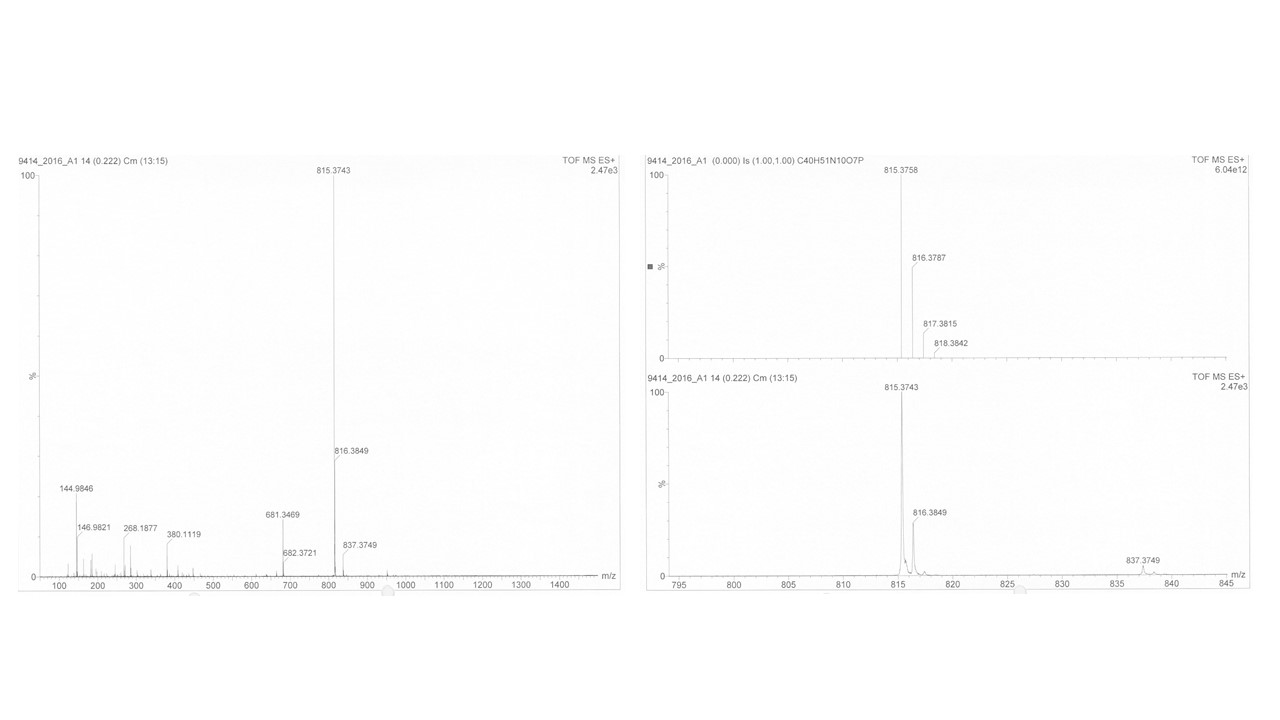


**Figure S8.** Inhibitor **38** HRMS spectra


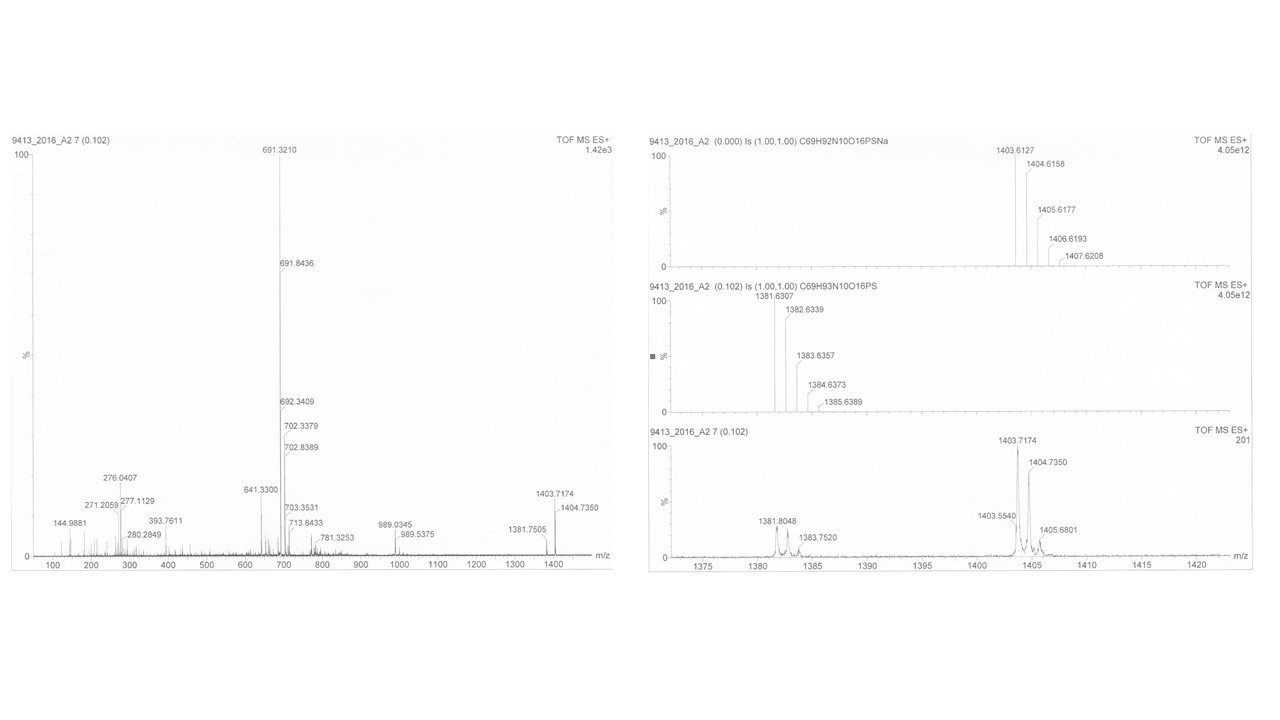
 **Figure S9.** HRMS spectra from coupling reaction of **35** with dipeptide Cbz-Lys(Boc)-Arg(Pbf)-OH


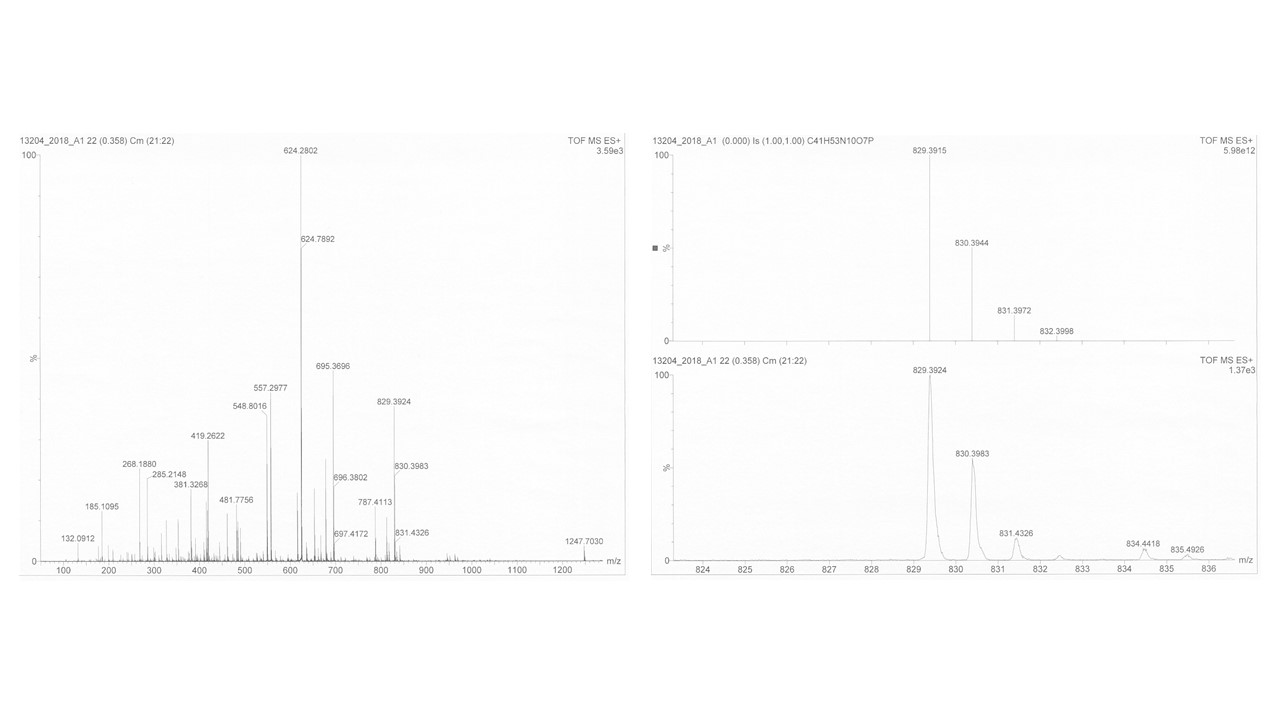


**Figure S10.** Inhibitor **39** HRMS spectra

**Supplementary References:**

1. Jackson D.S., Fraser S.A., Ni L.M., Kam C.M., Winkler U., Johnson D.A., Froelich C.J., Hudig D., Powers J.C.: Synthesis and evaluation of diphenyl phosphonate esters as inhibitors of the trypsin-like granzymes A and K and mast cell tryptase, J. Med. Chem., 1998, 41, 2289-2301.
2. Hamilton R., Walker B.J., Walker B.: A convenient synthesis of N-protected diphenyl phosphonate ester analogues of ornithine, lysine and homolysine, Tetrahedron Lett., 1993, 34, 2847-2850.
3. Oleksyszyn J., Subotkowska L., Mastalerz P.: Diphenyl 1-aminoalkanephosphonates, Synthesis, 1979, 985-986.
4. Sieńczyk M., Oleksyszyn J.: A convenient synthesis of new α-aminoalkylphosphonates, aromatic analogues of arginine as inhibitors of trypsin-like enzymes, Tetrahedron Lett., 2004, 45, 7251-7254.
5. Mancuso A., Swern D.: Activated dimethyl sulfoxide: useful reagents for synthesis, Synthesis, 1981, 165-185.
6. Burchacka E., Walczak M., Sieńczyk M., Dubin G., Zdżalik M., Potempa J., Oleksyszyn J.: The development of first Staphylococcus aureus SplB protease inhibitors: Phosphonic analogues of glutamine, Bioorg Med Chem Lett., 2012, 22, 5574-5578.
7. Joosens J., Van der Veken P., Lambeir A.-M., Augustyns K., Haemers A.: Development of irreversible diphenyl phosphonate inhibitors for urokinase plasminogen activator, J. Med. Chem., 2004, 47, 2411-5413.
8. Sieńczyk M., Oleksyszyn J.: A convenient synthesis of new α-aminoalkylphosphonates, aromatic analogues of arginine as inhibitors of trypsin-like enzymes, Tetrahedron Lett., 2004, 45, 7251-7254.
9. Oleksyszyn J., Boduszek B., Kam C.-M., Powers J.C.: Novel amidino-containing peptidyl phosphonates as irreversible inhibitors for blond coagulation and related serine proteases, J. Med. Chem., 1994, 37, 226-231.
10. Wendt M.D., Rockway T.W., Geyer A., McClellan W., Weitzberg M., Zhao X., Mantei R., Nienaber V.L., Stewart K.,Klinghofer V., Giranda V.L.: Identification of novel binding interactions in the development of potent, selective 2-naphthamidine inhibitors of urokinase. Synthesis, structural analysis, and SAR of N-phenyl amide 6-substitution, J. Med. Chem., 2004, 47, 303-324.
11. Magdolen P., Mačiarová M., Toma Š.: Ultrasound effect on the synthesis of 4-alkyl (aryl)aminobenzaldehydes, Tetrahedron, 2001, 57, 4781-4785.
